# Altered Levels of Plasma Inflammatory Cytokines and White Matter Integrity in Bipolar Disorder Patients With Suicide Attempts

**DOI:** 10.3389/fpsyt.2022.861881

**Published:** 2022-04-07

**Authors:** Xiaowei Jiang, Yingrui Guo, Linna Jia, Yue Zhu, Qikun Sun, Lingtao Kong, Feng Wu, Yanqing Tang

**Affiliations:** ^1^Brain Function Research Section, Department of Radiology, The First Affiliated Hospital of China Medical University, Shenyang, China; ^2^Department of Psychiatry, The First Affiliated Hospital of China Medical University, Shenyang, China; ^3^Department of Radiation Oncology, The First Affiliated Hospital of China Medical University, Shenyang, China; ^4^Department of Geriatric Medicine, The First Affiliated Hospital of China Medical University, Shenyang, China

**Keywords:** bipolar disorder, suicide attempts, inflammatory cytokine, diffusion tensor imaging, TNF-α, IL-1β, IL-6, white matter integrity

## Abstract

**Objective:**

Bipolar disorder (BD) has a higher lifetime rate of suicide attempts (SA) than other psychiatric disorders. Furthermore, BD patients with SA (BD + S) are prone to a worse quality of life. However, the pathophysiology of BD + S is poorly understood. To further reveal the potential mechanisms of BD + S, abnormalities in peripheral plasma inflammatory cytokines and brain white matter (WM) in BD + S, as well as the correlation between them are investigated.

**Methods:**

We tested the levels of TNF-α, IL-1β, and IL-6 in peripheral plasma and collected the diffusion tensor imaging (DTI) data from 14 BD + S, 24 BD patients without SA (BD-S), and 26 healthy controls (HCs). The three groups were matched by age and gender. The levels of TNF-α, IL-1β, and IL-6 were detected by Luminex multifactor detection technology, and the fractional anisotropy (FA) values were employed to depict the alterations of WM. Partial correlation analyses were conducted to detect correlations between levels of TNF-α, IL-1β, and IL-6 and changes of WM, and the relationships between severity of clinical symptoms, including scores of HAMD-17 and YMRS, and cytokine levels or FA values in all groups.

**Results:**

For plasma inflammatory cytokines, there was no significant difference in their levels except for IL-6 among the three groups. *Post-hoc* analyses revealed that increased IL-6 level was only detected in BD + S (*p* < 0.05, Bonferroni correction). For DTI, BD + S showed specifically decreased FA in the bilateral middle cerebellar peduncle and the left superior corona radiata compared to BD-S and HCs (*p* < 0.05, Bonferroni correction). Additionally, both BD + S and BD-S groups revealed decreased FA in the bilateral body and genu of corpus callosum (CC) compared to HCs (*p* < 0.05, Bonferroni correction). No significant correlation between plasma inflammatory cytokines and WM integrity was found. In the BD + S group, we found negative correlation between the scores of YMRS and FA values of the left middle cerebellar peduncle (*r* = −0.74, *p* = 0.035).

**Conclusion:**

The inflammation and impaired WM integrity may provide a scientific basis to understand the potential mechanisms of BD + S.

## Introduction

Among psychiatric disorders, bipolar disorder (BD) has the highest suicide incidence (1–4). A recent meta-analysis demonstrated that the lifetime prevalence of suicide attempts (SA) in BD was 33.9% (5), which is at least 20 times higher than the general population (6). Epidemiological studies also showed that approximately 20–60% of BD patients had attempted suicide in their lifetime, and approximately 4–19% died due to suicide (7). In addition, BD patients with SA (BD + S) have a worse quality of life (8) and are peculiarly prone to poor functional outcomes (9). However, the pathophysiology of suicide in BD continues to be poorly understood.

Increasing evidence suggests an important role of cell-mediated immune activation and chronic inflammation in the pathophysiology of BD and SA. For example, previous studies consistently showed both higher TNF-α and IL-6 levels were associated with BD (10–12). Similarly, abnormally higher levels of TNF-α, IL-1β, and IL-6 were shown in the SA population (13–15). However, limited research has focused on the immune system impairment of BD + S. To our knowledge, only one study has investigated plasma inflammatory cytokines of BD + S and found increased IL-1β expression (16). The above studies provide a perspective of immunology to understand BD + S.

Another common sign of abnormalities is in white matter (WM). Studies using diffusion tensor imaging (DTI), which is the commonly used neuroimaging method to investigate the impairment of WM (17), found that BD + S exhibited lower fractional anisotropy (FA) in the uncinate fasciculus (UF), the ventral frontal cortex, and the cerebellar regions than BD without SA (BD-S) and healthy controls (HCs) (18, 19). Additionally, lower FA in the orbital frontal cortex, the middle portion of the forceps minor, and the anterior and posterior portion of the right cingulum bundle were also found in BD + S compared to BD-S (20, 21). Furthermore, BD + S showed a smaller WM volume (22) and declined FA values in the corpus callosum (CC), which may be related to suicidality (23). In addition, increased white matter hyperintensities have been consistently reported in BD + S (24–27). In summary, BD + S may have the impairment of WM microstructure.

Moreover, peripheral plasma inflammatory cytokines are closely related to the aberrant WM (28–30). They could pass the blood-brain barrier to activate microglia, which may impair brain cells, including the myelin sheath – an essential component of WM (17). Although evidence has suggested the important roles of plasma inflammatory cytokines and WM impairment in BD + S, the complicated and interwoven relationship between plasma inflammatory cytokines and WM integrity in BD + S has rarely been investigated. Therefore, we combined the plasma levels of inflammatory cytokines with the WM microstructure method to explore the immunologic and neuroimaging changes in BD + S and the relationship between them to further reveal the potential mechanisms of BD + S.

## Materials and Methods

### Subjects

Sixty-four subjects aged from 15 to 47 years old were included in the study. Among them, 14 were BD + S, 24 were BD-S, and 26 were HCs; the three groups were age and gender matched. BD patients were recruited from the Department of Psychiatry, the First Affiliated Hospital of China Medical University. HCs were recruited by advertisement. All subjects were provided with written informed consent after a detailed description of the study. If they were juveniles, further consent was provided by their parents/legal guardians. The study was authorized by the Institutional Review Board of the First Affiliated Hospital of China Medical University.

For adult patients, the Structured Clinical Interview for Diagnostic and Statistical Manual of Mental Disorders, Fourth Edition (DSM-IV) Axis I Disorders (SCID-I) was used to determine whether the patients met the criteria of BD (31). For adolescent and child patients, the Schedule for Affective Disorders and Schizophrenia for School-Age Children-Present and Lifetime Version (K-SADS-PL) was used to determine whether the patients met the criteria of BD (32). All the BD patients were free of any other Axis I and Axis II disorders. HCs did not have a history of Axis I or Axis II disorders themselves or in their first-degree relatives. The severity of clinical symptoms was evaluated by using the 17-item Hamilton Rating Scale for Depression (HAMD-17) (33) and the Young Mania Rating Scale (YMRS) (34).

In the study, SA was defined as a self-destructive act with the purpose to die at least one attempt in one’s lifetime (35). A self-made scale based on the definition of SA and excluded the self-injurious behavior without suicidal purpose. The reason for exclusion of the self-injurious behavior is that it may be a confounder to obscure the potential mechanism of SA.

Assessment of diagnosis, severity of symptoms, and SA were completed by at least one researcher who had passed a consistency test for clinical assessment.

Subjects were excluded if they were on anti-inflammatory medications or had any somatic diseases which may cause potential brain structural changes such as neurological disorders, uncontrolled hypertension, uncontrolled diabetes, substance or alcohol abuse, a history of head trauma resulting in more than 5 min of unconsciousness, or any magnetic resonance imaging (MRI) contraindications. All the subjects underwent a general physical examination which showed no evidence of ongoing infection.

### Plasma Inflammatory Cytokines Analysis

Blood samples were collected between 10:00 a.m. and 2:00 p.m., with EDTA as an anticoagulant, following standard procedures. After centrifugation at 2,000 rpm for 10 min, the plasma samples were kept at −80°C for further analysis. The plasma inflammatory cytokine levels were measured by the immunoassay (Human Magnetic Luminex Assay, Human Premixed Multi-Analyte Kit, R&D Systems, Inc., Minneapolis, MN, United States). In this process, a Human magnetic premixed microparticle cocktail of antibodies (Kit Lot Number L120614) was used to magnetically label the samples.

### Magnetic Resonance Imaging Acquisition

MRI data were collected by the GE Signa HDX 3.0T scanner at the Department of Radiology, the First Affiliated Hospital of China Medical University. DTI scanning was performed using the following parameters: TR/TE = 17,000/86 ms, field of view = 24 cm × 24 cm, imaging matrix = 120 × 120, slice number = 65, slice thickness = 2 mm, slice spacing = 2 mm, acquired along 26 directions (25 with *b* = 1,000 s/mm^2^ and 1 with *b* = 0), and voxel size = 2 mm^3^. Subjects were told to close their eyes and relax while remaining awake throughout scanning.

### Image Processing

Pipeline for Analyzing braiN Diffusion imAges software^1^ was used to process DTI data. For each subject, the voxel-wise diffusion tensor matrix was first constructed in the native space. The eigenvalues and eigenvectors were then yielded by diagonalizing this matrix. Based on these three eigenvalues, each subject’s voxel-wise FA map was calculated. All FA maps were non-linearly registered to the FMRIB58_FA template and normalized to the Montreal Neurological Institute (MNI) space. After that, a mean FA map was generated for all subjects. Finally, FA maps were smoothed using a Gaussian filter kernel of 6 mm full width at half maximum.

### Statistical Analyses

Diffusion tensor imaging data were analyzed using SPM8.^2^ We performed a one-way analysis of covariance (ANCOVA) with age and sex as covariates to examine the statistical differences in WM tracts among three groups. Statistical significance was determined by voxel *p* < 0.005 and cluster *p* < 0.05 [Gaussian random field (GRF) correction]. The FA values for each cluster with statistical differences were extracted.

We used IBM SPSS Statistics (version 22.0, Armonk, NY, United States) for Windows to analyze the demographic and clinical characteristics of subjects. The independent-samples *t*-test was utilized to compare the duration of illness between BD + S and BD-S. One-way analysis of variance (ANOVA) was used to compare age, education years, total scores of HAMD-17 and YMRS, and FA values among BD + S, BD-S, and HCs. Additionally, Chi-square tests were adopted to compare differences in gender, disease state, medication status, and subcategories of BD. We utilized ANCOVA with age and sex as covariates to examine the significant differences in plasma inflammatory cytokine levels across the three groups. Statistical significance was determined by *p* < 0.05. Partial correlation analyses with age, sex, and medication as covariates were adopted to evaluate correlations between the FA values and levels of TNF-α, IL-1β, and IL-6, and the relationships between severity of clinical symptoms, including scores of HAMD-17 and YMRS, and cytokine levels or FA values in BD + S, BD-S, and HC group, respectively.

## Results

### Demographic Characteristics and Plasma Inflammatory Cytokine Levels

There were no significant differences in age, gender, and education years among BD + S, BD-S, and HCs. Duration of illness, medication status, disease state, and subcategories had no significant differences between the two subgroups of BD. The two patient subgroups showed significant higher scores of HAMD-17 and YMRS than HCs, and no significant differences were found between BD + S and BD-S (Table 1).

**TABLE 1:** Demographic characteristics and cytokines levels of subjects.

Variables	HC (*n* = 26)	BD + S (*n* = 14)	BD-S (*n* = 24)	*F/*χ*^2^/t*	*p*	*Post-hoc* comparison (Bonferroni), *p*		
						HC vs. BD + S	HC vs. BD-S	BD + S vs. BD-S
**Characteristics**								
Age, years	28.23 ± 7.69	26.79 ± 7.68	26.13 ± 1.91	0.41	0.67	1.00	1.00	1.00
Male/Female	10/16	6/8	12/12	0.68	0.71	NA	NA	NA
Education, years	15.44 ± 3.08	12.71 ± 2.58	13.54 ± 2.99	4.56	0.01*	0.02*	0.08	1.00
Duration of illness, months	NA	70.56 ± 80.64	39.71 ± 39.45	1.464	0.07	NA	NA	NA
Medication use	NA	11 (78.57%)	21 (87.50%)	0.53	0.65	NA	NA	NA
Antidepressants	NA	6 (42.86%)	14 (58.33%)	0.85	0.50	NA	NA	NA
Antipsychotics	NA	10 (71.43%)	11 (45.83%)	2.34	0.18	NA	NA	NA
Mood stabilizer	NA	9 (64.29%)	16 (66.67%)	0.02	1.00	NA	NA	NA
**Disease state**								
Depression	NA	4 (28.57%)	11 (45.83%)	1.10	0.29	NA	NA	NA
Mania/hypomania	NA	5 (35.71%)	3 (12.50%)	2.87	0.09	NA	NA	NA
Remission	NA	5 (35.71%)	10 (41.67%)	0.13	0.72	NA	NA	NA
**Subcategories of BD**								
BD-I	NA	9(64.29%)	10(41.67%)	1.81	0.18	NA	NA	NA
BD-II	NA	4(28.57%)	9(37.50%)	0.31	0.58	NA	NA	NA
Unclear	NA	1(7.14%)	5(20.83%)	1.25	0.26	NA	NA	NA
HAMD-17 scores	0.96 ± 1.54	11.39 ± 10.06	10.46 ± 10.74	10.88	0.00*	0.00*	0.00*	1.00
YMRS scores	0.04 ± 0.20	7.85 ± 9.67	5.08 ± 8.39	6.50	0.00*	0.00*	0.04*	0.73
**Cytokines, pg/ml**								
IL-1β	3.51 ± 1.16	3.70 ± 0.84	3.64 ± 1.00	0.81	0.45	0.75	0.69	1.00
IL-6	1.09 ± 0.19	1.34 ± 0.26	1.17 ± 0.22	6.02	0.01*	0.01*	0.53	0.047*
TNF-α	2.61 ± 0.68	2.82 ± 0.60	2.57 ± 0.72	0.87	0.43	0.83	1.00	0.87

Data are presented as number (%) or mean ± standard deviation; BD, bipolar disorder; n, number of subjects; HC, healthy control; BD + S, BD patients with suicide attempts; BD-S, BD patients without suicide attempts; HAMD-17, 17-item Hamilton Rating Scale for Depression; YMRS, Young Manic Rating Scale; *Significant level at p < 0.05; NA, not applicable.

**FIGURE 1:**
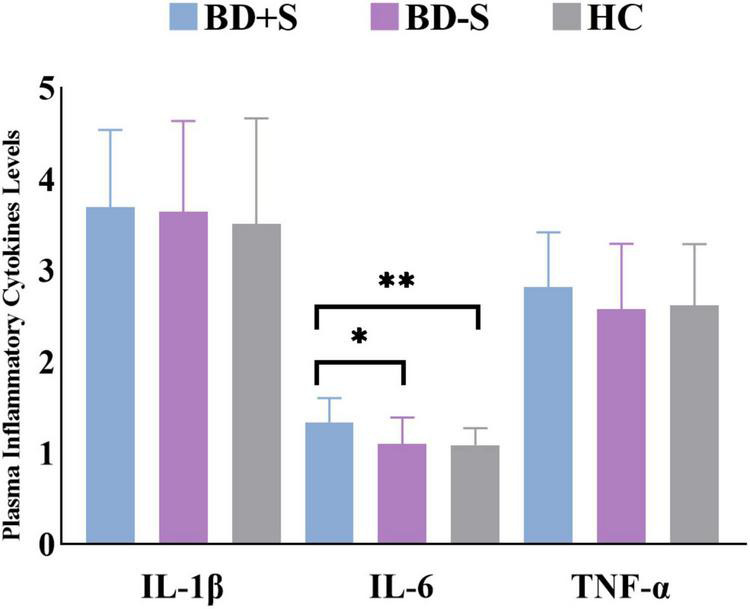
Levels of IL-1β, IL-6, and TNF-α in the BD + S, BD-S, and HC groups. ^∗∗^*p* < 0.01; ^∗^*p* < 0.05.

For plasma inflammatory cytokine levels, there was a significant difference in IL-6 level (*F* = 6.02, *p* = 0.01) but no significant difference in IL-1β level (*F* = 0.81, *p* = 0.45) or TNF-α level (*F* = 0.87, *p* = 0.43) among these three groups. *Post-hoc* analyses found significantly increased IL-6 level in BD + S compared to BD-S and HCs (*p* < 0.05, Bonferroni correction) (Figure 1 and Table 1).

### White Matter Integrity Findings

**FIGURE 2:**
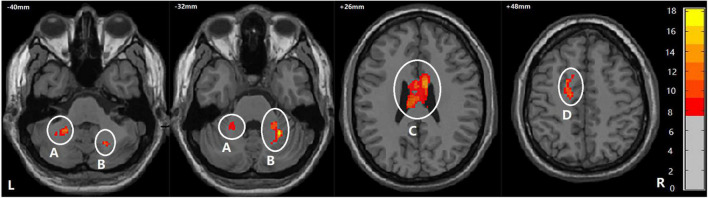
Regions showing WM differences among the BD + S, BD-S, and HC groups. A, the left middle cerebellar peduncle; B, the right middle cerebellar peduncle; C, the bilateral body and genu of corpus callosum; D, the left superior corona radiate. The number is z-coordinate; The color bar is the range of *F*-values; L, left; R, right.

**FIGURE 3:**
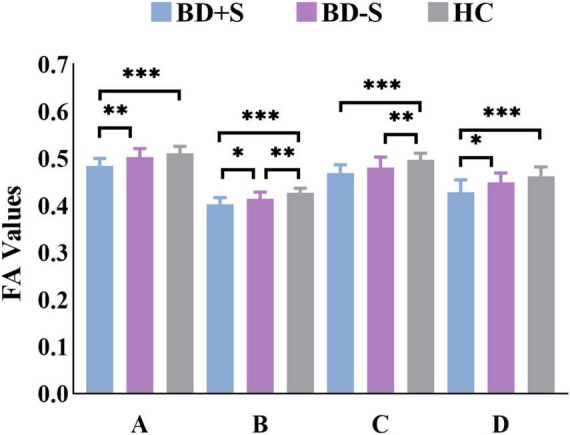
FA values of each cluster in the BD + S, BD-S, and HC groups. A, the left middle cerebellar peduncle; B, the right middle cerebellar peduncle; C, the bilateral body and genu of corpus callosum; D, the left superior corona radiate. ^∗∗∗^*p* < 0.001; ^∗∗^*p* < 0.01; ^∗^*p* < 0.05.

Among BD + S, BD-S, and HC groups, significant FA differences were found in the bilateral middle cerebellar peduncle, the bilateral body and genu of CC, and the left superior corona radiata (*p* < 0.005, GRF correction) (Figure 2 and Table 2). *Post-hoc* analyses revealed that compared to BD-S and HCs, BD + S showed significantly decreased FA in the bilateral middle cerebellar peduncle and the left superior corona radiata (*p* < 0.05, Bonferroni correction). Compared to HCs, both BD + S and BD-S groups showed significantly reduced FA in the bilateral body and genu of CC (*p* < 0.05, Bonferroni correction), and there were no significant differences between the two subgroups of BD (Figure 3).

**TABLE 2:** Differences of WM among BD + S, BD-S, and HC.

Cluster	Region	Voxels	Peak MNI coordinates			*F*-values*
			*x*	*y*	*z*	
A	Left middle cerebellar peduncle	122	−22	−48	−40	12.06
B	Right middle cerebellar peduncle	135	28	−50	−32	16.55
C	Bilateral body and genu of corpus callosum	701	8	2	26	11.77
D	Left superior corona radiate	112	−20	4	48	11.87

WM, white matter; MNI, Montreal Neurological Institute; *Significant at p < 0.005 by GRF correction.

### Correlation Analyses

#### Correlation Between the Plasma Inflammatory Cytokine Levels and the White Matter Integrity

No regions showed correlations between the plasma inflammatory cytokine levels and the FA values in all groups.

#### Correlation Between the Severity of Clinical Symptoms and Cytokine Levels

No correlations between the severity of clinical symptoms and cytokine levels were found in all groups.

#### Correlation Between the Severity of Clinical Symptoms and Fractional Anisotropy Values

**FIGURE 4:**
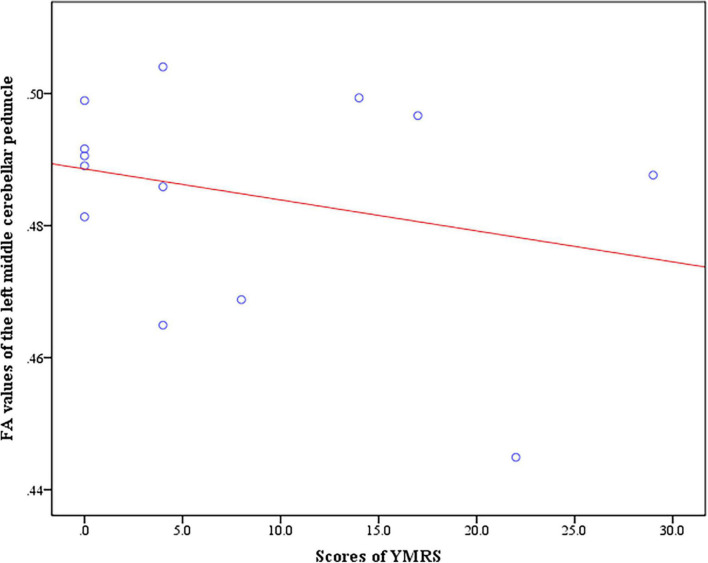
Negative correlation between the scores of YMRS and FA values of the left middle cerebellar peduncle in BD + S group.

In the BD + S group, we found negative correlation between the scores of YMRS and FA values of the left middle cerebellar peduncle (*r* = −0.74, *p* = 0.035) (Figure 4). And no correlation between the scores of HAMD-17 and FA values were found in BD + S group.

In the BD-S group, no correlations between the scores of HAMD-17 or YMRS and FA values were found.

## Discussion

The study explored alterations in the levels of TNF-α, IL-1β, and IL-6, as well as the WM integrity among BD + S, BD-S, and HCs. The correlations between the WM integrity and the levels of TNF-α, IL-1β, and IL-6 were also examined within each group. The findings revealed that BD + S exhibited specifically increased IL-6 levels and lower FA values in the bilateral middle cerebellar peduncle and the left superior corona radiate. Furthermore, both BD + S and BD-S showed decreased FA values in the bilateral body and genu of the corpus callosum, indicating the possible neuroimaging mechanisms of BD. However, no relationship between these plasma inflammatory cytokines and the WM integrity was found in any groups. What is more, we found that the FA values of left middle cerebellar peduncle and the score of YMRS were negatively correlated in BD + S.

Significantly elevated IL-6 level was specific to BD + S in this study. Accumulating evidence suggests that IL-6 is an important proinflammatory cytokine associated with BD (36–40) and suicide (41, 42). One study investigated a similar group found that BD patients with suicide risk had increased IL-1β levels (16). Inconsistent results were predominated in the BD patients and suicide population. For example, BD patients displayed increased levels of TNF-α and IL-1β compared to HCs (12, 16, 38, 40, 43). When comparing suicidal patients with non-suicidal ones and HCs, no significant difference in the levels of IL-6 and TNF-α was found (44). In contrast to the above studies, which focused just on BD or suicide, our research was concerned with SA in BD. Thus, these inconsistent results may be related to the heterogeneous groups. More studies investigating BD + S and BD-S are needed to confirm the results in the future. In addition, BD + S presented higher levels of IL-1β and TNF-α than BD-S and HCs, though it did not reach statistical significance. This may suggest that BD + S has a different immune pattern with BD-S and HCs, and the inflammation of BD + S is more aberrant than BD-S.

Regarding neuroimage, BD + S exhibited specifically impaired WM in the bilateral middle cerebellar peduncle and the left superior corona radiate. It is a pity that we did not find any evidence indicating that the above two brain regions are related to BD + S. The only study of BD + S reported that WM integrity impairments in the right cerebellar regions (19), which is similar with our results. This may indicate that the cerebellum plays a role in the mechanism of suicide in patients with BD. Since we did not find a direct correlation between corona radiata and BD + S, but we found indirect evidence that, internal capsule, which is anatomically connected with the corona radiata (45), is relevant to future SA in adolescents and young adults with BD (46). Although we did not find any alterations in FA values of internal capsule, we infer that the anatomical connection between corona radiata and internal capsule will lead to the functional interaction between them. Thus, corona radiata may be involved to the mechanism of BD + S through its relationship with internal capsule. Furthermore, Reich R. et al. found increased FA values of the corona radiata is positive correlated with greater impulsivity in BD + S (47), which may deepen understanding of our findings. Conversely, decreased FA values of corona radiata in BD + S was revealed in our finding. There are few studies on the WM integrity of corona radiata in BD + S. In the study of other diseases we observed increased FA values of corona radiata in patients with suicide attempts (48, 49). However, the FA values of corona radiata decreased in veterans with suicidal ideation (50). Therefore, the results of the FA values of corona radiata are contradictory in suicidal population, and further study is needed to confirm the role of corona radiata in BD + S.

The FA values of the bilateral body and genu of CC decreased in both BD + S and BD-S groups, indicating that the impaired bilateral body and genu of CC may be involved in the neuroimaging mechanism of BD. CC is the main interhemispheric connector that contains 300 million axons and connects most cortical regions of the brain. It is responsible for integrating motor, cognitive, sensory, and learning information between the two hemispheres (51). A growing body of literature suggests that the CC plays an important role in the pathophysiological mechanism of BD (52–54), and numerous DTI studies have demonstrated that BD patients showed impaired WM integrity in the CC compared to HCs (53, 55–58). Nery-Fernandes et al. found a reduction in the genu and isthmus area of CC in BD patients, but no difference in any subregion of CC between BD + S and BD-S (52), which corresponds to our results. Likewise, similar to the cytokine findings, we also found that the FA values decreased gradually from HC to BD-S to BD + S. Our findings may support the idea that impaired WM occur in a graded manner, i.e., BD + S > BD-S > HCs.

A relationship between the plasma inflammatory cytokines and the WM integrity was not found in the current study. By searching the literature, we found only one study investigating the relationship between WM and cytokines in BD, and the results revealed that the TNF-α level is inversely associated with WM integrity in BD-I patients (28). Nevertheless, the study did not include HCs. On the other hand, it is inconclusive whether the increased cytokine levels in the central nervous system are parallel to those in the peripheral blood (59). As a result, cytokines in peripheral blood may not reflect WM injury. Therefore, the relationship between them may not be determined. Another study, which drew a similar conclusion with ours, showed that the levels of IL-6 and TNF-α did not have a significant increase in BD patients compared to HCs (60). Relatively small number of studies limit our interpretation of the results. Consequently, we should pay attention to BD + S and provide more evidence for the underlying mechanism of the BD + S.

BD + S displayed negative correlation between the scores of YMRS and FA values of the left middle cerebellar peduncle, indicating that the decreased FA values of the left middle cerebellar peduncle is related to manic or hypomanic symptoms of BD + S. Similarly, Olivito G. et al. demonstrated that the cerebellum, which including the middle cerebellar peduncle, plays an important role in mania and hypomania in BD patients (61). The finding is novel and hint subgroup analysis may dig deeper into the relationship between the brain and symptoms.

### Limitation

We recruited BD patients of different ages, different disease states, and different medication use statuses. To explore the potential influence, we compared the FA values and IL-6 levels divided by age, state, and medication use in BD + S and BD-S groups. We found that there were no statistical differences between adolescent and adult groups, between the different states, and between the medication use and medication-free groups (Supplementary Tables 1–6). Therefore, age, state, and medication may have little influence on the results. However, the potential impact of these factors cannot be ignored. In addition, the study was a cross-sectional study with a small sample size, which requires a follow-up and larger sample sizes in the future.

## Conclusion

In summary, the results suggest that BD + S may present specific and much more aberrant immunologic and neuroimaging changes compared with BD-S. It may provide a scientific basis to understand the potential mechanisms of BD + S and calls for attention to the suicide attempts of BD patients.

## Data Availability Statement

The original contributions presented in the study are included in the article/Supplementary Material, further inquiries can be directed to the corresponding author.

## Ethics Statement

The studies involving human participants were reviewed and approved by the Medical Scientific Research Ethics Committee of the First Affiliated Hospital of China Medical University. Written informed consent to participate in this study was provided by the participants’ legal guardian/next of kin.

## Author Contributions

XJ was responsible for conceptualization, investigation, data curation, writing original draft, and funding acquisition. YG performed the data processing, statistical analyses, visualization, and wrote the original draft. LJ wrote the original draft. YZ validated the results. QS acquired the data. FW and LK recruited the patients, confirmed the diagnosis, and acquired the funding. YT was responsible for conceptualization, project administration, and funding acquisition. All authors reviewed and approved the manuscript.

## Conflict of Interest

The authors declare that the research was conducted in the absence of any commercial or financial relationships that could be construed as a potential conflict of interest.

## Publisher’s Note

All claims expressed in this article are solely those of the authors and do not necessarily represent those of their affiliated organizations, or those of the publisher, the editors and the reviewers. Any product that may be evaluated in this article, or claim that may be made by its manufacturer, is not guaranteed or endorsed by the publisher.

## Footnotes

1
http://www.nitrc.org/projects/panda

2
www.fil.ion.ucl.ac.uk/spm/software/spm8
