# Supplementary material for: Altered Levels of Plasma Inflammatory Cytokines and White Matter Integrity in Bipolar Disorder Patients With Suicide Attempts
Source: Front Psychiatry. 2022 Apr 7;13:861881. doi: 10.3389/fpsyt.2022.861881 (PMC9021603; doi:10.3389/fpsyt.2022.861881)
Supplement: Supplementary file 1 [file Data_Sheet_1.docx]

**Supplementary tables**

| Table 1. The comparison of FA values and IL-6 levels between adolescent and adult patients in BD+S group. | | | | |  |
| --- | --- | --- | --- | --- | --- |
| Cluster | Adolescent patients (age<18)  (n=2) | Adult patients (age≥18)  (n=12) | *t* | *p* | |
| A | 0.48±0.02 | 0.48±0.02 | 0.01 | 0.99 | |
| B | 0.41±0.02 | 0.40±0.01 | 0.49 | 0.64 | |
| C | 0.47±0.03 | 0.47±0.02 | 0.04 | 0.97 | |
| D | 0.42±0.01 | 0.43±0.03 | 0.27 | 0.79 | |
| IL-6 | 1.21±0.27 | 1.36±0.27 | 0.72 | 0.49 | |
| Note: Data are presented as mean ± standard deviation; ^*^ Significant level at *p*<0.05. | | | | | |

| Table 2. The comparison of FA values and IL-6 levels between adolescent and adult patients in BD-S group. | | | | |
| --- | --- | --- | --- | --- |
| Cluster | Adolescent patients (age<18)  (n=2) | Adult patients (age≥18)  (n=22) | *t* | *p* |
| A | 0.50±0.01 | 0.50±0.02 | 0.27 | 0.79 |
| B | 0.41±0.00 | 0.41±0.02 | 0.01 | 0.99 |
| C | 0.47±0.00 | 0.48±0.02 | 0.37 | 0.71 |
| D | 0.45±0.01 | 0.45±0.02 | 0.28 | 0.78 |
| IL-6 | 1.26±0.04 | 1.14±0.16 | 0.96 | 0.35 |
| Note: Data are presented as mean ± standard deviation; * Significant level at *p*<0.05. | | | | |

| Table 3. The comparison of FA values and IL-6 levels among depression, mania or hypomania, and remission patients in BD+S group. | | | | | |  |
| --- | --- | --- | --- | --- | --- | --- |
| Cluster | Depression patients  (n=4) | Mania or hypomania patients  (n=5) | Remission patients  (n=5) | *F* | *p* | |
| A | 0.48±0.02 | 0.48±0.02 | 0.49±0.01 | 0.78 | 0.48 | |
| B | 0.40±0.00 | 0.40±0.1 | 0.41±0.02 | 0.26 | 0.78 | |
| C | 0.47±0.01 | 0.46±0.02 | 0.47±0.02 | 0.20 | 0.82 | |
| D | 0.43±0.02 | 0.42±0.02 | 0.43±0.04 | 0.08 | 0.93 | |
| IL-6 | 1.27±0.31 | 1.35±0.29 | 1.39±0.25 | 0.16 | 0.85 | |
| Note: Data are presented as mean ± standard deviation; ^*^ Significant level at *p*<0.05. | | | | | | |

| Table 4. The comparison of FA values and IL-6 levels among depression, mania or hypomania, and remission patients in BD-S group. | | | | | |
| --- | --- | --- | --- | --- | --- |
| Cluster | Depression patients  (n=11) | Mania or hypomania patients  (n=3) | Remission patients  (n=10) | *F* | *p* |
| A | 0.51±0.02 | 0.51±0.02 | 0.50±0.02 | 0.91 | 0.42 |
| B | 0.41±0.01 | 0.42±0.00 | 0.41±0.02 | 0.01 | 0.99 |
| C | 0.48±0.02 | 0.49±0.02 | 0.48±0.02 | 0.33 | 0.72 |
| D | 0.45±0.02 | 0.46±0.01 | 0.45±0.02 | 0.10 | 0.91 |
| IL-6 | 1.22±0.13 | 1.26±0.04 | 1.07±0.17 | 2.90 | 0.08 |
| Note: Data are presented as mean ± standard deviation; ^*^ Significant level at *p*<0.05. | | | | | |

| Table 5. The comparison of FA values and IL-6 levels between medication use and medication-free patients in BD+S group. | | | | |
| --- | --- | --- | --- | --- |
| Cluster | Medication use patients  (n=11) | Medication-free patients  (n=3) | *t* | *p* |
| A | 0.48±0.01 | 0.48±0.01 | 0.08 | 0.94 |
| B | 0.40±0.02 | 0.40±0.01 | 0.41 | 0.69 |
| C | 0.47±0.02 | 0.47±0.01 | 0.51 | 0.62 |
| D | 0.43±0.03 | 0.43±0.02 | 0.22 | 0.83 |
| IL-6 | 1.29±0.24 | 1.50±0.33 | 1.24 | 0.24 |
| Note: Data are presented as mean ± standard deviation; ^*^ Significant level at *p*<0.05. | | | | |

| Table 6. The comparison of FA values and IL-6 levels between medication use and medication-free patients in BD-S group. | | | | |
| --- | --- | --- | --- | --- |
| Cluster | Medication use patients  (n=21) | Medication-free patients (n=3) | *t* | *p* |
| A | 0.50±0.02 | 0.50±0.02 | 0.80 | 0.44 |
| B | 0.41±0.02 | 0.41±0.01 | 0.37 | 0.71 |
| C | 0.47±0.01 | 0.48±0.02 | 0.45 | 0.66 |
| D | 0.45±0.02 | 0.45±0.02 | 0.27 | 0.79 |
| IL-6 | 1.15±0.08 | 1.15±0.17 | 0.02 | 0.98 |
| Note: Data are presented as mean ± standard deviation; ^*^ Significant level at *p*<0.05. | | | | |
